# Impact of renal denervation on cardiac remodeling in resistant hypertension: A meta‐analysis

**DOI:** 10.1002/clc.24222

**Published:** 2024-01-29

**Authors:** Linfeng Xie, Yuanzhu Li, Suxin Luo, Bi Huang

**Affiliations:** ^1^ Department of Cardiology The First Affiliated Hospital of Chongqing Medical University Chongqing China

**Keywords:** cardiac remodeling, renal denervation, resistant hypertension

## Abstract

Twelve studies involving 433 patients were included. After RDN treatment, LVMI decreased by 13.08 g/m^2^ (95% confidence interval [CI]: −18.38, −7.78; *p* < .00001), PWTd decreased by 0.60 mm (95% CI: −0.87, −0.34; *p* < .00001), IVSTd decreased by 0.78 mm (95% CI: −1.06, −0.49; *p* < .00001), and LVEF increased by 1.80% (95% CI: 0.71, 2.90; *p* = .001). However, there were no statistically significant improvements in LVIDd (95% CI: −1.40, 0.24; *p* = .17) and diastolic function (E/A) (95% CI: −0.04, 0.14; *p* = .28). Drug treatment for resistant hypertension (RH) is challenging. Renal denervation (RDN) is one of the most promising treatments for RH. Although studies have shown RDN can control blood pressure, the impacts of RDN on cardiac remodeling and cardiac function are unclear. This meta‐analysis evaluated the effect of RDN on cardiac structure and function in patients with RH. PubMed, Embase, and Cochrane were used to conduct a systematic search. The main inclusion criteria were studies on patients with RH who received RDN and reported the changes in echocardiographic parameters before and after RDN. Echocardiographic parameters included left ventricular mass index (LVMI), end‐diastolic left ventricular internal dimension (LVIDd), left ventricular end‐diastolic posterior wall thickness (PWTd), end‐diastolic interventricular septum thickness (IVSTd), E/A, and left ventricular ejection fraction (LVEF). Data was analyzed using RevMan. Twelve studies involving 433 patients were included. After RDN treatment, LVMI decreased by 13.08g/m2 (95%confidence interval [CI]: −18.38, −7.78, *p* < .00001), PWTd decreased by 0.60mm (95% CI: −0.87, −0.34, *p* < 0.00001), IVSTd decreased by 0.78mm (95% CI: −1.06, −0.49, *p* < .00001), and LVEF increased by 1.80% (95% CI: 0.71, 2.90, *p* = .001). However, there were no statistically significant improvements in LVIDd (95% CI: −1.40, 0.24, *p* = .17) and diastolic function (E/A) (95% CI: −0.04, 0.14, *p* =.28). This meta‐analysis finds that RDN can improve left ventricular hypertrophy and ejection fraction in patients with RH but has no significant effect on LVIDd and diastolic function. However, more studies are warranted due to the lack of a strict control group, a limited sample size, and research heterogeneity.

## Graphical abstract

Resistant hypertension (RH) is associated with higher risk of target organs damage and the treatment of RH with medicine is challenging. Denervation (RDN) is a minimally invasive method to lower blood pressure by ablating renal sympathetic nerves; however, the impacts of RDN on cardiac structure and function are unclear. This meta‐analysis found that RDN can improve left ventricular hypertrophy and left ventricular ejection fraction in patients with RH

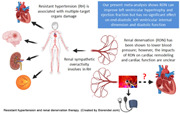

## 1 INTRODUCTION

Resistant hypertension (RH) refers to the persistence of blood pressure above the target despite the simultaneous use of three optimal tolerable doses of antihypertensive drugs, including a diuretic.^1^ The prevalence of RH is estimated at 10%–20% in the hypertensive population.^2^ Patients with RH have a 50% higher risk of developing adverse cardiovascular events compared with well‐controlled hypertension.^3^ More importantly, RH contributes to left ventricular (LV) hypertrophy, which is an independent risk factor for cardiovascular mortality. Meanwhile, RH can lead to LV dysfunction, including systolic and diastolic dysfunction.^4^ Previous studies have shown that the incidence of LV hypertrophy in RH is about 55%–75% detected by echocardiography and 91% of LV high voltage on electrocardiogram (ECG).^5^

In the past decade, renal denervation (RDN), a minimally invasive procedure, was introduced to treat RH. This approach addresses the challenges of achieving target blood pressure among patients with RH using drug treatment alone, considering the role of renal nerves (sympathetic nerves) in blood pressure regulation.^6^ The principle of RDN for treating hypertension is based on attenuating sympathetic signaling to the kidneys by ablating both the afferent and the more abundant efferent sympathetic nerves adjacent to the renal arteries percutaneously using various forms of energy such as radiofrequency and ultrasound. This ablation leads to the reduction or even elimination of the effects of overactive renal sympathetic activity on blood pressure.^7^ Previous studies have demonstrated RDN as a promising strategy for patients with RH, and current guidelines and consensus recommend that RDN may be a safe and effective method in patients with RH.^8^, ^9^, ^10^, ^11^ Although RDN can reliably lower blood pressure, its influence on the cardiac structure and function is inconsistent in different studies due to relatively small sample size, high heterogeneity of enrolled patients, and differences in operating methods and experience among different centers.^12^, ^13^, ^14^, ^15^, ^16^, ^17^, ^18^, ^19^, ^20^, ^21^, ^22^, ^23^ Therefore, this meta‐analysis evaluated the effect of RDN on cardiac remodeling in patients with RH undergoing RDN therapy.

## 2 METHODS

### 2.1 Literature search

A systematic search was conducted in PubMed, Embase, and Cochrane with the search terms “renal denervation,” “echocardiogram,” and “echocardiography” to retrieve all published literature from January 2012 to November 2022. The keywords were searched according to the PICOS principle. To avoid omissions, we performed a manual search of the references of relevant articles.

### 2.2 Eligibility criteria and study screening

The final included studies should met the following criteria: (1) patients diagnosed with RH and received RDN therapy and (2) reported echocardiographic data on LV structure and function before and after RDN therapy including LV mass index (LVMI), end‐diastolic LV internal dimension (LVIDd), LV end‐diastolic posterior wall thickness (PWTd), end‐diastolic interventricular septum thickness (IVSTd), E/A, and LV ejection fraction (LVEF). Reviews, conference summaries, unrelated topics, non‐English literature, and studies without corresponding study endpoints were excluded. Two researchers independently conducted preliminary screening based on titles and abstracts, and then the full text was assessed if it met the inclusion criteria for the present study. The quality evaluation of the included studies was conducted by the NOS scale^24^ and was divided into three levels: low (<5 points), medium (5–8 points), and high quality (8–9 points).

### 2.3 Study endpoints and data extraction

The endpoints associated with LV structure were LVMI, LVIDd, PWTd, and IVSTd. The endpoints associated with LV function were LVEF and E/A. For each study, the following information was extracted: the first author, publication year, study design, study population, age, gender, body mass index, comorbidities, antihypertensive drugs, follow‐up time, and echocardiographic parameters before and after RDN therapy.

### 2.4 Statistical analysis

Study endpoints were all continuous variables and expressed as mean ± standard deviation. According to the recommendations of the Cochrane Collaboration and Preferred Reporting Items for Systematic Reviews and Meta‐Analyses guidelines, statistical analysis was conducted using Review Manager (RevMan) version 5.4. The effect of RDN on LV structure and function was expressed with combined mean and 95% confidence interval (CI). The heterogeneity of the study was represented by *I* ^2^ and *p* values. *p* > .10 or *I* ^2^ < 50 was considered to indicate low heterogeneity, and a fixed‐effects model was used. Otherwise, heterogeneity was considered to be high, and a random‐effects model was used. Furthermore, a sensitivity analysis was performed by removing one study conducted on RH with chronic kidney disease. Subgroup analysis was performed based on the follow‐up time. In addition, because the RDN technology was innovated in 2015, subgroup analysis was conducted by studies published before and after 2015. Subgroup analysis was also conducted based on the presence or absence of a control group. Publication bias in the meta‐analysis was assessed using funnel plot.

## 3 RESULTS

### 3.1 Eligible studies

**Figure 1:**
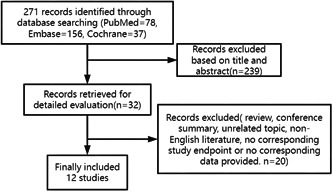
Flow diagram of the study selection process.

As shown in Figure 1, 12 studies involving 13 cohorts and 433 patients were included. Table 1 summarizes the characteristics of the included studies. All studies were prospective, and the included patients had RH; only one study had patients with RH with chronic kidney disease. Three studies contained a control group.^12^, ^17^, ^23^ All studies had moderate NOS quality grades. The sample size ranged from 13 to 66 patients. Among the 433 patients, 253 (58.4%) were male, 42.9% had diabetes, 27.8% had coronary heart disease, 7.0% had atrial fibrillation, 64.0% had dyslipidemia, and 13.0% had stroke. All patients underwent RDN therapy while taking antihypertensive drugs, with an average of 4.64 antihypertensive drugs. Follow‐up duration in these studies was either 6 or 12 months.

**Table 1:** Clinical baseline characteristics of patients enrolled in the included studies.

Study	Year	Study design	Control group	Patients	RDN (*n*)	Age	Gender (male/female)	BMI (kg/m^2^)	Diabetes	Dyslipidemia
Almeida et al.	2016	Prospective	No	RH	31	65 ± 7	15/16	31.8 ± 5.5	22	21
Brandt et al.	2012	Prospective	Yes	RH	46	63.1 ± 10.2	31/15	28.6 ± 3.4	21	32
Dores et al.	2014	Prospective	No	RH	34 (22^a^)	62.7 ± 7.6	17/17	30.9 ± 5.3	22	23
Feyz et al.	2017	Prospective	No	RH	31	64 ± 10	15/16	29 ± 4	8	22
Gao1 et al.	2021	Prospective	No	RH	30	55.93 ± 10.94	22/8	–	14	16
Gao2 et al.	2021	Prospective	No	RH	30	62.73 ± 12.50	22/8	–	16	13
Kiuchi et al.	2016	Prospective	No	RH with CKD	45	53.9 ± 11.3	26/19	30.2 ± 4.3	15	–
Luo et al.	2022	Prospective	Yes	RH	13	55 ± 15	6/7	26.84 ± 3.49	7	10
McLellan et al.	2015	Prospective	No	RH	14	64 ± 9	10/4	31 ± 3	2	10
Palionis et al.	2016	Prospective	No	RH	15	54 ± 7.2	8/7	34.46 ± 3.46	4	11
Ripp et al.	2015	Prospective	No	RH	60	54.6 ± 9.5	33/27	32.9 ± 6.2	–	–
Schirmer et al.	2014	Prospective	No	RH	66	63.5 ± 1.2	36/30	29.4 ± 0.6	23	42
Tsioufis et al.	2015	Prospective	Yes	RH	18	56 ± 10	12/6	33.6 ± 5.4	6	10

Study	Coronary heart disease	Atrial fibrillation	Stroke	Antihypertensive drugs	Follow‐up (month)	Interesting endpoints
Almeida et al.	10	1	2	5.8 ± 1.1	12	LVMI, LVIDd, PWTd, IVSTd, LVEF, E/A
Brandt et al.	20	7	8	4.7 ± 0.5	1, 6	LVMI, LVIDd, PWTd, IVSTd, LVEF, E/A
Dores et al.	7	–	3	5.8 ± 1.0	6	LVMI, LVEF, E/A
Feyz et al.	16	2	–	4 ± 1	6	LVMI, LVIDd, PWTd, IVSTd, LVEF, E/A
Gao1 et al.	6	3	6	3.13 ± 1.04	12	LVIDd, PWTd, IVSTd, LVEF
Gao2 et al.	10	1	2	3.73 ± 1.20	12	LVIDd, PWTd, IVSTd, LVEF
Kiuchi et al.	6	2	6	4.7 ± 1.2	6	LVMI, LVIDd, PWTd, IVSTd, LVEF
Luo et al.	–	–	–	5.23 ± 1.01	12	LVIDd, PWTd, IVSTd, LVEF
McLellan et al.	2	2	3	4.9 ± 1.8	6	LVMI, LVIDd, PWTd, IVSTd, LVEF
Palionis et al.	4	–	–	6.3 ± 1.1	6	LVMI, LVIDd, PWTd, IVSTd, E/A
Ripp et al.	–	–	–	–	6	LVMI, LVIDd, PWTd, IVSTd, LVEF, E/A
Schirmer et al.	14	–	–	4.3 ± 0.1	6	E/A
Tsioufis et al.	–	–	–	4.5 ± 0.6	6	LVMI, LVIDd, PWTd, IVSTd, LVEF, E/A

*Note*: Continuous variables are expressed as mean ± SD or median (interquartile range).

Abbreviations: BMI, body mass index; CKD, chronic kidney disease; IVSTd, end‐diastolic interventricular septum thickness; LVEF, left ventricular ejection fraction; LVIDd, end‐diastolic left ventricular internal dimension; LVMI, left ventricular mass index; PWTd, left ventricular end‐diastolic posterior wall thickness; RH, resistant hypertension.

^a^ Number of patients who completed follow‐up.

### 3.2 Antihypertensive effects of RDN

Three studies^13^, ^18^, ^23^ reported a change in antihypertensive medications after RDN therapy. A significantly reduced antihypertensive medication was observed after RDN treatment (−1.26; 95% CI: −1.63, −0.89; *p* < .00001). One study showed that the types of antihypertensive drugs decreased by 1.1 (*p* < .00001).^19^ Eleven studies^12^, ^13^, ^14^, ^15^, ^16^, ^17^, ^18^, ^20^, ^21^, ^22^, ^23^ reported blood pressure levels after RDN treatment. Both systolic blood pressure and diastolic blood pressure decreased after RDN treatment (systolic blood pressure: −22.27, 95% CI: −23.95, −20.59; *p* < .00001; diastolic blood pressure: −8.14, 95% CI: −9.20, −7.07; *p* < .00001).

### 3.3 Effect of RDN on LV structure

**Figure 2:**
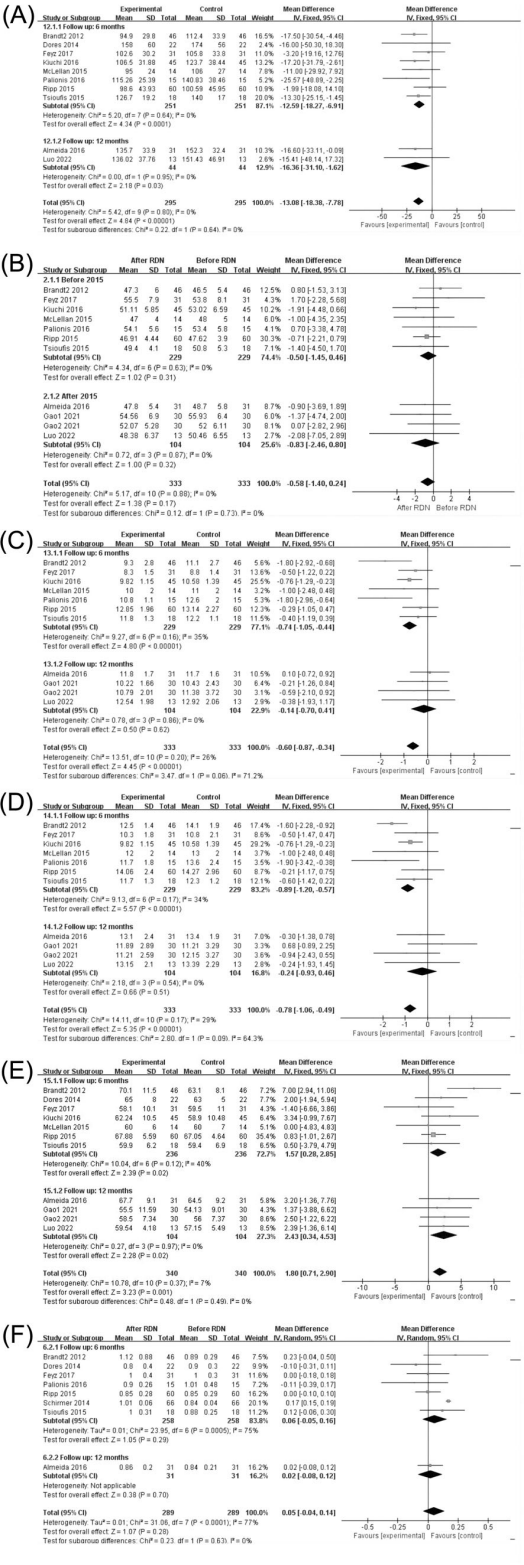
Effect of renal denervation (RDN) on cardiac structure and function in resistant hypertension (RH) patients. (A) Effect of RDN on left ventricular mass index (LVMI) in RH patients. (B) Effect of RDN on end‐diastolic left ventricular internal dimension (LVIDd) in RH patients. (C) Effect of RDN on left ventricular end‐diastolic posterior wall thickness (PWTd) in RH patients. (D) Effect of RDN on end‐diastolic interventricular septum thickness (IVSTd) in RH patients. (E) Effect of RDN on left ventricular ejection fraction (LVEF) in RH patients. (F) Effect of RDN on E/A in RH patients.

Nine studies with 295 patients provided LVMI at baseline and at the end of follow‐up. Eight studies followed up participants for 6 months, and the duration of follow‐up was 12 months in one study. The results of the meta‐analysis are shown in Figure 2A. LVMI showed a statistically significant decrease by 12.59 g/m^2^ (95% CI: −18.27, −6.91; *p* < .0001) after 6 months of follow‐up and by 16.36 g/m^2^ (95% CI: −31.10, −1.62; *p* = .03) after 12 months of follow‐up.

Ten studies provided LVIDd, PWTd, and IVSTd values at baseline and at the end of follow‐up. Seven studies followed up participants for 6 months, and three studies followed up participants for 12 months. The meta‐analysis results of LVIDd are shown in Figure 2B. There was no significant change in LVIDd before and after RDN therapy after 6 months (95% CI: −1.45, 0.46; *p* = .31) or 12 months of follow‐up (95% CI: −2.46, 0.80; *p* = .32). The meta‐analysis results of PWTd are shown in Figure 2C. After 6 months of follow‐up, PWTd decreased by 0.74 mm (95% CI: −1.05, −0.44; *p* < .00001), but there was no statistically significant change after 12 months of follow‐up (95% CI: −0.70, 0.41; *p* = .62). The meta‐analysis results of IVSTd are shown in Figure 2D. After 6 months of follow‐up, IVSTd decreased by 0.89 mm (95% CI: −1.20, −0.57; *p* < .00001). However, there was no statistically significant change after 12 months of follow‐up (95% CI: −0.93, 0.46; *p* = .51).

### 3.4 Effect of the RDN on LV function

Ten studies provided baseline data and follow‐up results on LVEF. Participants were followed up for 6 months and 12 months in seven and three studies, respectively. The meta‐analysis results are shown in Figure 2E. LVEF increased by 1.57% after 6 months (95% CI: 0.28, 2.85; *p* = .02) and 2.43% after 12 months of follow‐up (95% CI: 0.34, 4.53; *p* = .02).

Eight studies provided baseline data and follow‐up results on E/A. Seven studies followed up participants for 6 months, and three studies followed up participants for 12 months. The meta‐analysis results are shown in Figure 2F. There was no statistically significant change in E/A before and after RDN therapy after 6 months (95% CI: −0.05, 0.16; *p* = .29) or 12 months (95% CI: −0.08, 0.12; *p* = .70).

### 3.5 Subgroup analysis

#### 3.5.1 Subgroup analysis by publication year

The included studies were divided into two subgroups before and after 2015 because the RDN technology was innovated around 2015. The effects of RDN therapy on LVMI, PWTd, IVSTd, and LVEF in the two subgroups were compared (Figure S1). The results of the meta‐analysis of studies published before 2015 showed that the change in LVMI was −11.43 g/m^2^ (95% CI: −17.60, −5.26; *p* = .0003), PWTd −0.73 mm (95% CI: −1.16, −0.29; *p* = .001), IVSTd −0.91 mm (95% CI: −1.30, −0.52; *p* < .00001), and LVEF 2.30% (95% CI: 1.02, 3.57; *p* = .0004). The results of the meta‐analysis of studies published after 2015 showed that the change in LVMI was −14.31 g/m^2^ (95% CI: −22.46, −6.17; *p* = .0006), PWTd −0.58 mm (95% CI: −0.90, −0.25; *p* = .0005), IVSTd −0.64 mm (95% CI: −1.01, −0.27; *p* = .0008), and LVEF 2.15% (95% CI: 0.37, 3.92; *p* = .02).

#### 3.5.2 Subgroup analysis based on with or without a control group

The results of the meta‐analysis without a control group are shown in Figure S2. The results showed that the change in LVMI was −11.70 g/m^2^ (95% CI: −18.50, −4.91; *p* = .0007), PWTd −0.56 mm (95% CI: −0.86, −0.26; *p* = .0002), IVSTd −0.62 mm (95% CI: −0.97, −0.27; *p* = .0004), and LVEF 1.36% (95% CI: 0.12, 2.61; *p* = .03). The meta‐analysis results of studies with a control group showed that the change in LVMI was −13.34 g/m^2^ (95% CI: −20.46, −6.21; *p* = .0002), PWTd −0.87 mm (95% CI: −1.40, −0.33; *p* = .002), IVSTd −1.00 mm (95% CI: −1.42, −0.57; *p* < .00001), and LVEF 4.26% (95% CI: 2.38, 6.13; *p* < .00001).

### 3.6 Sensitivity analysis

After excluding the study by Kiuchi et al.,^19^ in whose study the participants had RH and chronic kidney disease, the change in LVMI was −12.45 g/m^2^ (95% CI: −18.14, −6.76; *p* < .00001), PWTd −0.55 mm (95% CI: −0.86, −0.24; *p* = .0005), IVSTd −0.79 mm (95% CI: −1.12, −0.45; *p* < .0001), and LVEF 1.70% (95% CI: 0.57, 2.83; *p* = .003). The changes in LVIDd (95% CI: −1.30, 0.44; *p* = .33) and E/A (95% CI: −0.04, 0.14; *p* = .28) were not statistically significant.

### 3.7 Publication bias

**Figure 3:**
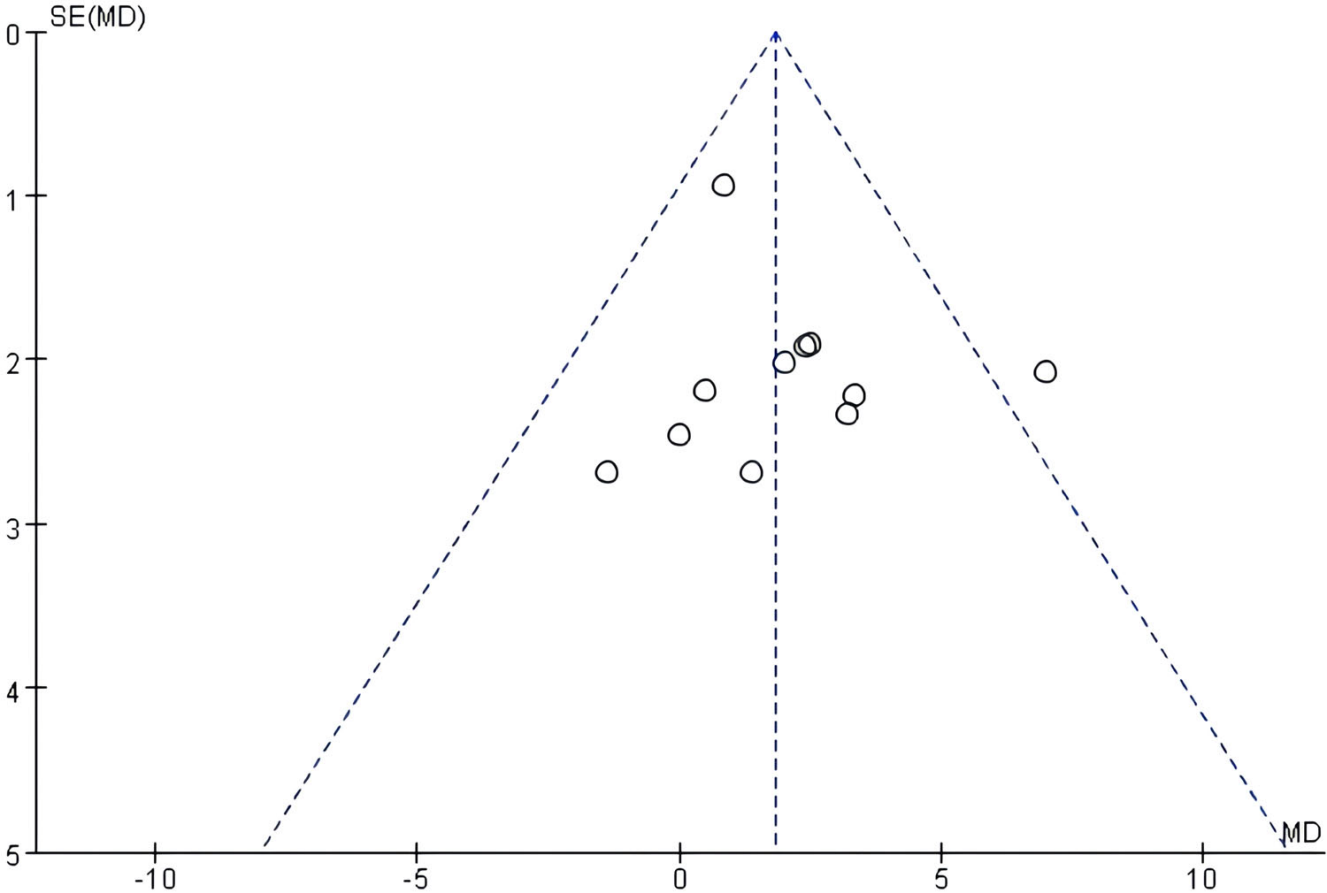
Funnel plot.

Publication bias was assessed using a funnel plot (Figure 3). The funnel plot was symmetrical, suggesting low publication bias.

## 4 DISCUSSION

Chronic activation of the sympathetic nervous system is involved in the occurrence, progression, and maintenance of hypertension,^25^ and sympathetic overactivity has long been considered as a major factor in the occurrence of RH.^26^ Meanwhile, excessive activity of the sympathetic nervous system is important in the development of cardiac remodeling in hypertensive patients.^27^, ^28^ There is evidence that the sympathetic nervous system mediates hypertension‐induced ventricular hypertrophy by directly stimulating the adrenergic receptor of cardiomyocytes.^29^

As early as the 1980s, research showed that renal sympathectomy could reduce the blood pressure level and improve the symptoms related to severe hypertension. However, due to the limited methods at that time, several adverse reactions occurred after renal sympathectomy, limiting its widespread application.^30^ Unlike surgical resection of the renal sympathetic nervous system, RDN ablates the afferent and efferent nerves of the renal sympathetic nervous system through a minimally invasive approach. RDN reduces renal sympathetic efferent activity, manifested by a significant decrease in norepinephrine secretion.^31^ RDN can also reduce the activation of the sympathetic nervous system throughout the whole body, as renal sympathetic afferent nerves play an important role in stimulating the activity of the hypothalamic sympathetic nervous system.^32^ Moreover, studies have shown that besides its role in treating RH, RDN may also be effective in the management of other diseases associated with excessive activation of the sympathetic nervous system, such as congestive heart failure,^33^ atrial fibrillation,^34^ obstructive sleep apnea syndrome.^35^ Therefore, the benefits of RDN may be pleiotropic, including attenuating overactivation of the sympathetic nervous system, lowering blood pressure, and improving cardiac arrhythmias, and so forth.

This meta‐analysis focused on the effects of RDN on cardiac structure and function in RH patients, mainly based on the following three reasons. First, cardiac hypertrophy is prevalent in patients with RH. Eleven cross‐sectional and longitudinal studies, including 3325 patients, revealed that the incidence of echocardiographic LVH ranged from 55% to 75%.^5^ Second, previous studies have shown that the reduction of peripheral blood pressure was not the true endpoint in judging the prognosis of hypertensive patients; it was only a surrogate endpoint. Moreover, the reduction in peripheral blood pressure did not directly lead to the parallel reduction in cardiovascular morbidity and mortality.^36^ Conversely, intermediate endpoints such as ventricular hypertrophy were shown to have reliable associations with cardiovascular outcomes.^37^, ^38^ Ventricular hypertrophy is an important manifestation of target organ damage related to hypertension. Muiesan et al.^39^ found that the inappropriate increase in LV mass in hypertensive patients was independently related to the occurrence of cardiovascular events, and the prognosis of hypertensive patients with improved LV hypertrophy was relatively good.^37^, ^38^ In addition, although cardiac magnetic resonance imaging is more accurate than echocardiography in assessing cardiac structure and function,^40^, ^41^ echocardiography is more commonly used in clinical practice. Therefore, we focused on the changes in echocardiographic parameters before and after RDN therapy. Kordalis et al.^42^ also evaluated the improvement of target organs in RH patients who received RDN using meta‐analysis. In addition to LVMI and E/A, Kordalis also observed changes in vascular indicators such as central enhancement index and pulse wave conduction velocity. Our results were consistent with the main findings from Kordalis, but we focused more on the changes in cardiac structure and function, especially the thickness of the ventricular septum and LV posterior wall, which are the most important indicators of cardiac remodeling. Furthermore, we performed subgroup analysis according to the follow‐up time, which further illustrated the long‐term effects of RDN on cardiac structure and function.

This meta‐analysis showed that RDN can improve cardiac structure and function in RH patients. In terms of LV structure, RDN can improve LVMI, PWTd, and IVSTd. For LV function, RDN can improve the LVEF. It was worth noting that compared with 6 months of follow‐up after RDN, after 12 months of follow‐up, some indicators, such as PWTd and IVSTd, were not statistically different, which may be related to sympathetic nerve regeneration and central sympathetic feedback regulation.^22^ Therefore, it is necessary to extend the observation time to determine the long‐term effects of RDN on cardiac structure and function in RH patients. Moreover, our meta‐analysis confirms that the blood pressure of RH patients was lower, and the number of antihypertensive medications taken by RH patients decreased after RDN treatment. In terms of safety, two included studies described safety outcomes, and no significant complications occurred.^19^, ^22^ In addition, the security of RDN has been confirmed by a large number of studies and recommended as a secure method by guidelines.^8^, ^9^, ^10^, ^11^ Taken together, RDN is a safe and effective way to lower blood pressure and improve cardiac remodeling.

## 5 LIMITATIONS

There were some limitations in this study. First, this was a meta‐analysis of observational studies. Although there was no statistical heterogeneity, there was heterogeneity in the included patients, operation experience, and operational details, thus inevitably causing systematic bias. Second, it was impossible to assess the long‐term effects of RDN on cardiac structure and function in RH patients because all of the studies had a short follow‐up duration. Third, all patients used antihypertensive drugs during RDN treatment; most studies did not set a sham surgery control group and only compared the changes in cardiac structure and function before and after RDN therapy. Therefore, the effects of blood‐pressure reduction or antihypertensive drugs leading to changes in cardiac structure and function cannot be excluded. Finally, due to the limitations in study design and ethical concerns, there are no studies comparing the efficacy of RDN treatment alone with antihypertensive drug treatment alone. Therefore, further studies are needed to clarify the impact of RDN on cardiac structure and function in patients with RH.

## 6 CONCLUSION

RDN can improve LV hypertrophy and LVEF in RH patients but has no significant effect on LVIDd and diastolic function. However, due to the lack of a strict control group, a limited sample size, and study heterogeneity, more studies are still warranted to clarify the impact of RDN on cardiac remodeling in patients with RH.

## CONFLICT OF INTEREST STATEMENT

The authors declare no conflict of interest.

## Supporting information


**Supplemental Figure 1**. Subgroup analysis based on the publication year (A: Effect of RDN on LVMI in RH patients; B: Effect of RDN on PWTd in RH patients; C: Effect of RDN on IVSTd in RH patients; D: Effect of RDN on LVEF in RH patients). RDN, renal denervation; RH, resistant hypertension; LVMI, left ventricular mass index; PWTd, left ventricular end‐diastolic posterior wall thickness; IVSTd, end‐diastolic interventricular septum thickness; LVEF, left ventricular ejection fraction.

**Supplemental Figure 2**. Subgroup analysis based on with or without control group (A: Effect of RDN on LVMI in RH patients; B: Effect of RDN on PWTd in RH patients; C: Effect of RDN on IVSTd in RH patients; D: Effect of RDN on LVEF in RH patients). RDN, renal denervation; RH, resistant hypertension; LVMI, left ventricular mass index; PWTd, left ventricular end‐diastolic posterior wall thickness; IVSTd, end‐diastolic interventricular septum thickness; LVEF, left ventricular ejection fraction.

## Data Availability

Data sharing not applicable to this article as no datasets were generated or analyzed during the current study.
