# Supplementary figures and images for: Impact of renal denervation on cardiac remodeling in resistant hypertension: A meta‐analysis
Source: Clin Cardiol. 2024 Jan 29;47(2):e24222. doi: 10.1002/clc.24222 (PMC10823454; doi:10.1002/clc.24222)

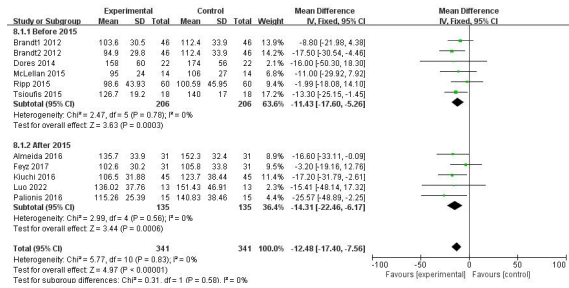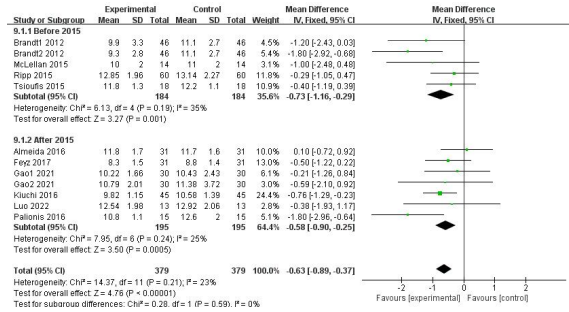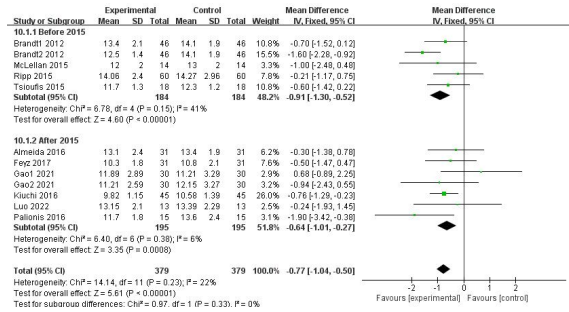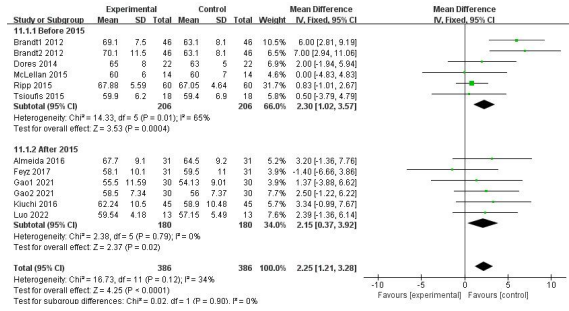

Supplement: Supplementary file 1 — Supplemental Figure 1. Subgroup analysis based on the publication year (A: Effect of RDN on LVMI in RH patients; B: Effect of RDN on PWTd in RH patients; C: Effect of RDN on IVSTd in RH patients; D: Effect of RDN on LVEF in RH patients). RDN, renal denervation; RH, resistant hypertension; LVMI, left ventricular mass index; PWTd, left ventricular end‐diastolic posterior wall thickness; IVSTd, end‐diastolic interventricular septum thickness; LVEF, left ventricular ejection fraction. [file CLC-47-e24222-s001.pdf]

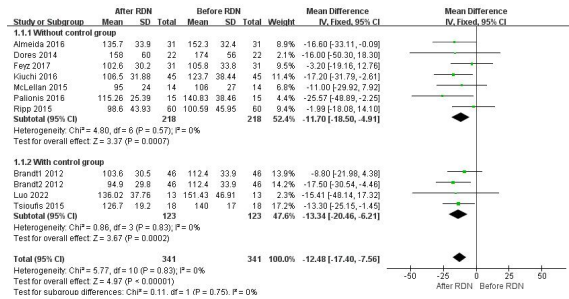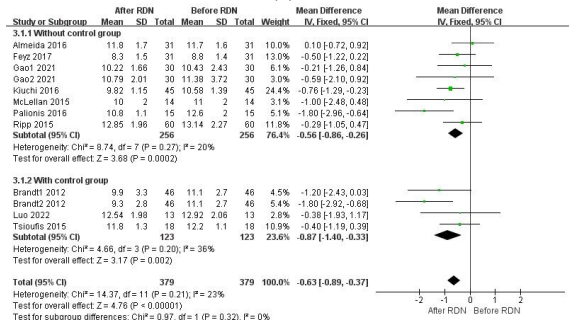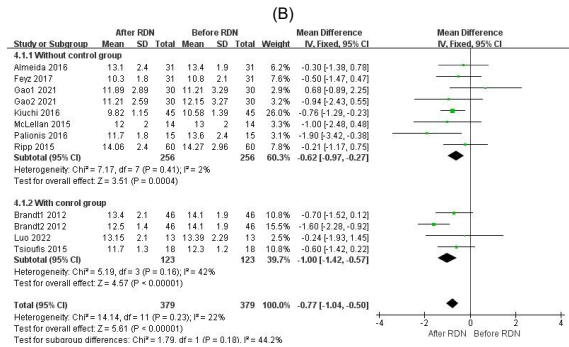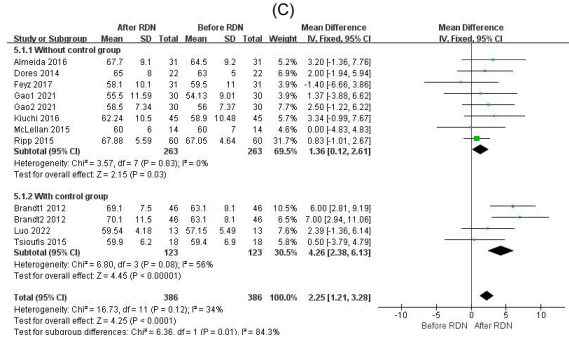

Supplement: Supplementary file 2 — Supplemental Figure 2. Subgroup analysis based on with or without control group (A: Effect of RDN on LVMI in RH patients; B: Effect of RDN on PWTd in RH patients; C: Effect of RDN on IVSTd in RH patients; D: Effect of RDN on LVEF in RH patients). RDN, renal denervation; RH, resistant hypertension; LVMI, left ventricular mass index; PWTd, left ventricular end‐diastolic posterior wall thickness; IVSTd, end‐diastolic interventricular septum thickness; LVEF, left ventricular ejection fraction. [file CLC-47-e24222-s002.pdf]
